# Influence of Different Types, Utilization Times, and Volumes of Aging Barrels on the Metabolite Profile of Red Wine Revealed by ^1^H-NMR Metabolomics Approach

**DOI:** 10.3390/molecules28186716

**Published:** 2023-09-20

**Authors:** Suwanan Denchai, Suppached Sasomsin, Cheunjit Prakitchaiwattana, Thanitaporn Phuenpong, Kunaporn Homyog, Wanwimon Mekboonsonglarp, Sarn Settachaimongkon

**Affiliations:** 1 Program in Biotechnology, Faculty of Science, Chulalongkorn University, Bangkok 10330, Thailand; 6370195223@student.chula.ac.th; 2 Department of Food Technology, Faculty of Science, Chulalongkorn University, Bangkok 10330, Thailand; 3 Innovation & Winemaking Division, Siam Winery Company Limited, Samut Sakhon 74000, Thailand; suppached.s@siamwinery.com; 4 Center of Veterinary Diagnosis, Faculty of Veterinary Science, Mahidol University, Nakhon Pathom 73170, Thailand; 5 Scientific and Technological Research Equipment Center (STREC), Chulalongkorn University, Bangkok 10330, Thailand; 6 Emerging Processes for Food Functionality Design Research Unit, Chulalongkorn University, Bangkok 10330, Thailand; 7 Omics Sciences and Bioinformatics Center, Faculty of Science, Chulalongkorn University, Bangkok 10330, Thailand

**Keywords:** red wine, oak aging, metabolite, metabolome, foodomics, omics, Thailand

## Abstract

It is well recognized that the aging process is a critical step in winemaking because it induces substantial chemical changes linked to the organoleptic properties and stability of the finished wines. Therefore, this study aimed to investigate the influence of different types, utilization times, and volumes of aging barrels on the metabolite profile of red wines, produced from Thai-grown Shiraz grapes, using a non-targeted proton nuclear magnetic resonance (^1^H-NMR) metabolomics approach. As a result, 37 non-volatile polar metabolites including alcohols, amino acids, organic acids, carbohydrates and low-molecular-weight phenolics were identified. Chemometric analysis allowed the discrimination of wine metabolite profiles associated with different types of aging containers (oak barrels vs. stainless-steel tanks), as well as the utilization times (2, 6 and >10 years old) and volumes (225, 500 and 2000 L) of the wooden barrels employed. Significant variations in the concentration of formate, fumarate, pyruvate, succinate, citrate, gallate, acetate, tyrosine, phenylalanine, histidine, γ-aminobutyrate, methionine and choline were statistically suggested as indicators accountable for the discrimination of samples aged under different conditions. These feature biomarkers could be applied to manipulate the use of aging containers to achieve the desired wine maturation profiles.

## 1. Introduction

Wine is a fermented beverage resulted from alcoholic fermentation of grape extracts or must by the activity of selected yeast strains, with occasionally malolactic fermentation, followed by maturation, stabilization, clarification and packaging [1]. Traditionally, major wine producing regions are restricted to temperate zones between 30 and 50 degrees of latitude where both the famous “old world” wine producers, e.g., France, Spain, Italy, and “new world” wine producers, e.g., USA, Australia, South Africa, Chile and Argentina are located [1]. During the last three decades, wine production has widely been expanded into nontraditional regions, even those with tropical and subtropical conditions [2]. In Thailand, wine grape plantations and industrial winemaking have been established, experimented with and developed since the 1990s [3]. As part of a modern lifestyle, wine has become a sign of social status and an increasingly marketable beverage commodity [4]. To date, more than five well-established wineries can be found in the northern and central part of Thailand where wines from both Thai indigenous and French grape varieties are mainly produced [3]. It should be mentioned that the reputation and uniqueness of Thai wines have progressively gained attention from both domestic and international consumers [2]. 

From a molecular viewpoint, wine is regarded as a complex beverage system consisting of hundreds of biomolecules, including sugars, proteins, amino acids, organic acids, phenolic compounds, pectic substances, alcohols, esters, carbonyls, as well as other varietal and aroma volatiles, present in a wide range of concentrations [1]. Changes in the chemical composition of wine are known to be predominantly influenced by both environmental and human factors related to geographic origin, viticultural practice and oenological processes which play a determining role in the quality, sensory attributes and typicality of the finished wine products [1]. Several attempts aiming at characterizing and validating the chemical identity, also called molecular authenticity, of wines in association with their geographical indications, grape varieties, fermentation and maturation techniques as well as adulteration issues have been extensively documented [5,6]. Among analytical approaches and technical strategies found in the literature, metabolomics is one of the tools most recently acknowledged in wine authenticity and traceability determination [5,7]. 

Metabolomics, a comprehensive characterization of low-molecular-weight metabolites (usually <1.5 kDa) present in complex biological systems [8], has been implemented and has provided novel insights in the area of wine science [7,9]. Thanks to advanced analytical instruments, the application of nuclear magnetic resonance (NMR) and mass spectrometry (MS)-based metabolomics for molecular characterization and authentication of wines linked to specific viticultural and oenological traits are extensively documented [5,7,10]. From a technological point of view, changes in the wine metabolome influenced by particular fermentation and maturation processes are the two major research domains that have considerably received attention. The impacts of genetic traits, physiological adaptation and metabolic activities of yeasts and bacterial starters on the formation of metabolites in wine during alcoholic and malolactic fermentation have been widely reported [11,12,13]. Apart from this, dynamic changes in the metabolite profiles of wine influenced by post-fermentation treatments have also been unraveled [4], with particular attention on different wine maturation and aging techniques [14,15,16,17,18]. It should be mentioned that progressive developments in wine metabolomics have opened new opportunities to assess the vinification process from a more holistic perspective to ensure wine quality and traceability [4]. Nevertheless, to our best knowledge, information on wine metabolomics studies in new latitude winegrowing regions is practically limited.

Therefore, the objectives of this study were to characterize and compare non-volatile polar metabolite profiles of red wines, made from Shiraz grapes grown in Thailand, using a non-targeted proton nuclear magnetic resonance (^1^H-NMR) metabolomics approach. The metabolite fingerprints of wine samples aged under different conditions were statistically compared by means of multivariate analysis. Finally, potential biomarker metabolites responsible for the discrimination of different types, utilization times and volumes of aging containers used in the maturation process of samples are proposed.

## 2. Results and Discussion

### 2.1. Acidity and Major Chemical Composition of Wine Samples

The acidity and major chemical composition of wine samples are presented in Table 1. Results demonstrated that the pH and TA values of samples ranged between 3.55–3.69 and 6.15–6.80 g tartaric acid/L, respectively. The alcoholic strength and residual sugar contents of samples ranged between 13.29–15.54%vol and 5.35–7.44 g glucose/L, respectively. Although some significant differences (*p* ≤ 0.05) in pH, TA, alcoholic strength and residual sugar content were noticeable among samples, no trends responsible for differentiating the type of aging containers could be observed for these parameters. The total phenolic contents of samples ranged between 1582.22 and 2673.89 mg GAE/L with significant variations within the three replicates and among groups of samples (*p* ≤ 0.05). It was remarkable that wines aged in stainless-steel tanks and old oak barrels had significantly lower total phenolic contents compared to those found in samples aged with new and medium-used oak barrels (*p* ≤ 0.05). 

It is well-recognized that oak aging is one of the most influential factors on the abundance of phenolic compounds present in red wines [18,19]. A significantly higher in total phenolic content of red wines aged in wooden barrels compared to the ones aged in stainless-steel tanks has been documented [20,21]. One of the key explanations could be that many phenolic substances, such as gallic acid, ellagic acid, coumarins and ellagitannins, are substantially extracted and released from oak woods into the wine during barrel aging [18]. Regardless of oak wood extraction, stainless-steel tanks contain a certain amount of metal ions which can react with wine polyphenols and accelerate oxidation resulting in a decrease in the concentration of these compounds in the finished product [20]. Nevertheless, the quantity of phenolic compounds and their rate of extraction generally diminish with the utilization of the barrels over successive years [22]. Our results are in agreement with the literature that reported a decrease in phenolics derived from oak woods with an increased barrel utilization time [22,23,24]. It has been acknowledged that the presence of phenolic compounds, at an appropriate level, contributes significantly to the flavor complexity and mouthfeel characteristics of red wines [22]. Therefore, the selection of aging container material is another factor that should be carefully considered in order to attain a desired wine characteristic and style.

### 2.2. ^1^H-NMR Data Acquisition and Metabolite Identification

**Figure 1:**
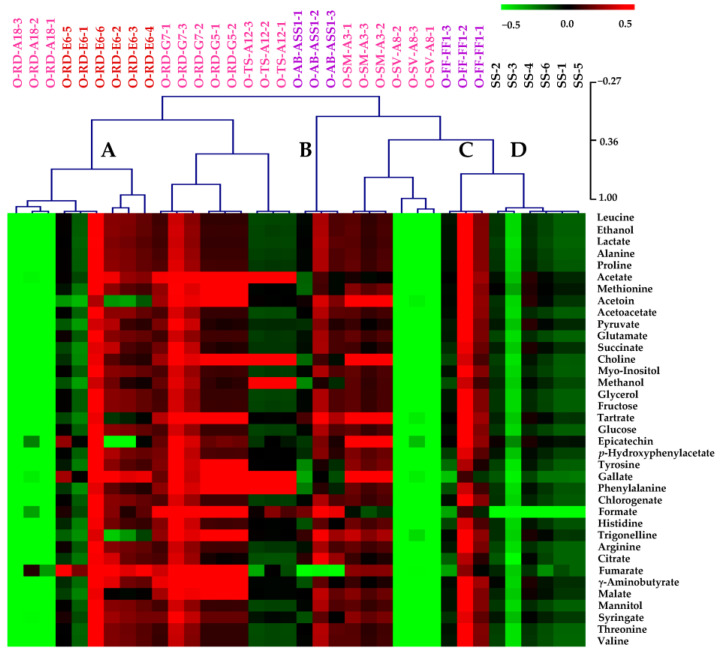
Heatmap visualization and hierarchical clustering of ^1^H-NMR-derived non-volatile polar metabolite profiles of Thai Shiraz wine samples aged in stainless-steel tanks (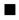), new (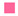), medium-used (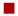) and old (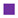) oak wood barrels. The dendrogram represents sample clusters based on Pearson’s correlation coefficient with average linkage. Each square in the heatmap expresses normalized metabolite content within the color range. The red color indicates a higher content of the corresponding metabolite compound. For interpretation of the color range used in this figure, the reader is referred to the relative quantification of metabolites in Supplementary Table S2.

A representative one-dimensional ^1^H-NMR spectrum of a Thai Shiraz wine sample with an assigned peak number corresponding to the positions of metabolites and their chemical shift assignment are presented in Figure S1 and Table S1, respectively. Results revealed that major metabolites present in grape must and wine, e.g., glucose, fructose, tartrate, malate, citrate, succinate, lactate, acetate, gallate and epicatechin [1], could be simultaneously detected and quantified using a non-targeted ^1^H-NMR metabolomics approach. A total of 37 metabolites including alcohols, amino acids, organic acids, sugars, carbohydrate derivatives, and low-molecular-weight phenolic compounds present in wine samples could be successfully identified in this study (Figure 1). This list of compounds corresponded well with those previously identified in red wines using a high-resolution ^1^H-NMR spectrometer (500–600 MHz) elsewhere [25,26,27,28,29]. Recently, a number of publications have demonstrated that ^1^H-NMR-based metabolomics is an effective tool to monitor dynamic changes in the wine metabolite profile, also called the metabolite fingerprint or metabolome, influenced by various oenological practices [7,10,30]. The advantages of using an ^1^H-NMR spectroscopic technique are its relatively simple sample preparation, short analysis times, good reproducibility and adequate specificity [7]. In this study, a non-targeted ^1^H-NMR analysis was applied to attain a global overview of the molecular fingerprints of Shiraz wine samples. Usually, multivariate statistics are performed after ^1^H-NMR measurement for classification of samples based on the variations in their metabolite profiles [30]. According to our previous work, the sum of ^1^H-NMR signal intensities (arbitrary units) from all bins accountable for individual metabolites were normalized against the TSP internal standard, median centering and log scaling before subjecting them to chemometric analysis [31]. HCA and PLS-DA were performed for discrimination of the metabolite profiles among wine samples by means of non-supervised and supervised pattern recognition, respectively. 

### 2.3. Overall Comparative Metabolite Profiling of Wine Samples

Heatmap visualization combined with hierarchical cluster analysis was performed to evaluate the overall relationships among ^1^H-NMR metabolite profiles of Shiraz wine samples matured under different conditions (*n* = 36) (Figure 1). This non-supervised pattern recognition technique aims to identify metabolite patterns without any a priori knowledge of the data [30]. Results demonstrated that wines matured in stainless-steel tanks were obviously grouped into a separated cluster (cluster D) from samples aged in oak wood barrels based on their metabolite profiles. Within the group of oak-aged wines, samples were classified into different clusters according to the types of barrels used in their maturation process. A good distinction could be observed for samples aged in medium-used (cluster A) and old barrels (cluster B and C) whereas the metabolite patterns of wines aged in new barrels were comparatively diverse. Besides sample clustering revealed by the dendrogram, different colors in the heatmap indicate the relative quantification of respective metabolites. The red color indicates a higher abundance, and the green color indicates a lower abundance of metabolites among samples [32]. It could be seen that most metabolites were present in the relatively higher abundances in red wines that underwent aging in oak wood barrels compared to the ones matured in stainless-steel tanks. For interpretation of the colors used in Figure 1, the integrated ^1^H-NMR signal intensities expressed in log_10_ transformed [arbitrary unit] of metabolites were statistically compared by means of one-way ANOVA and presented in Supplementary Table S2.

### 2.4. Influence of Aging Container Type on the Metabolite Profiles of Wine Samples

**Figure 2:**
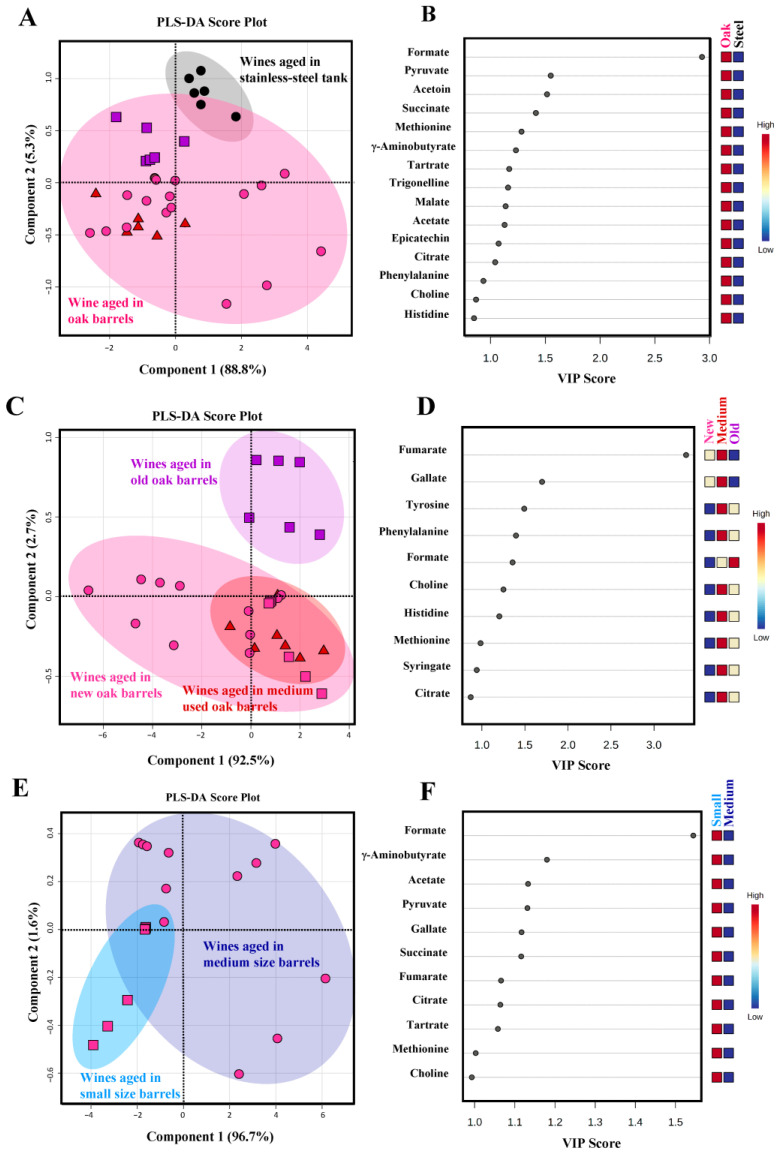
PLS-DA score plots (panel (**A**,**C**,**E**)) and VIP scores (panel (**B**,**D**,**F**)) for the comparison of ^1^H-NMR-derived non-volatile polar metabolite profiles of Thai Shiraz wine samples aged in stainless-steel tanks (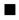), new (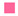), medium-used (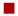) and old (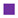) oak wood barrels. Squares in the VIP score panel express normalized non-volatile polar metabolite content within the color range. The red color indicates a higher content of the corresponding compound.

Supervised pattern recognitions by PLS-DA were performed to discriminate the ^1^H-NMR metabolite profiles of Shiraz wine samples aged under different conditions (Figure 2). Unlike HCA, supervised multivariate analysis applies predefined treatment or sample group information, i.e., material type, utilization time and size of aging containers in this study, to build a classification model followed by statistical testing and model validation [30]. To compare the overall metabolite profiles among samples (*n* = 36), a PLS-DA score plot was constructed with a prediction accuracy of 80.56%, *R*^2^ = 0.587 and *Q*^2^ = 0.483 (Figure 2A). Results demonstrated a good separation between wine samples aged in stainless-steel tanks and oak wood barrels. VIP scores with a value greater than 1.0 suggested that the concentrations of most indicative metabolites accountable for the discrimination, i.e., formate, pyruvate, acetoin, succinate, methionine, γ-aminobutyrate, tartrate, trigonelline, malate, acetate, epicatechin and citrate, were relatively higher in wine samples aged in oak wood barrels compared to the ones matured in stainless-steel tanks (Figure 2B). This was in accordance with the results from cluster analysis, as mentioned above, indicating a significant impact of wooden-barrel aging on the higher abundances of metabolites present in the wines.

Several attempts to characterize and compare the metabolite composition between barrel-aged and non-barrel-aged wines have been documented [15,21,33]. Our results revealed the effectiveness of ^1^H-NMR metabolomics for the differentiation of wine metabolome related to different types of aging container material. This observation was in agreement with the works of Lv et al. [33] and Maioli et al. [21] who also found significantly higher abundances of various metabolites in wooden-barrel-aged wines compared to the ones matured in other kinds of containers. As already discussed for the total phenolic content, a large variety of compounds including both aroma volatile and non-volatile metabolites can be extracted from oak woods and transferred into the wine during barrel aging [18]. This phenomenon essentially contributes to a progressive development in the sensorial characteristics of the finished wine products. Nevertheless, it should be mentioned that the use of oak wood barrels for wine aging involves a high economic cost. Therefore, wine maturation in stainless-steel tanks combined with the addition of oak chips or oak staves has been recently applied as an alternative method to introduce desirable oak flavor characteristics into wines [22]. 

Apparently, the PLS-DA score plot in Figure 2A showed that the distribution pattern of wine samples aged in oak wood barrels (displayed in the red circle) seemed to correspond somehow with the age of barrels used in their maturation process. To provide more insights into the impact of barrel attributes on the metabolite profiles of red wines in this study, two separated PLS-DA analyses were performed for comparison of samples aged in oak wood barrels with differences in their utilization time and volume, respectively. 

### 2.5. Influence of Barrel Age on the Metabolite Profiles of Wine Samples 

**Figure 3:**
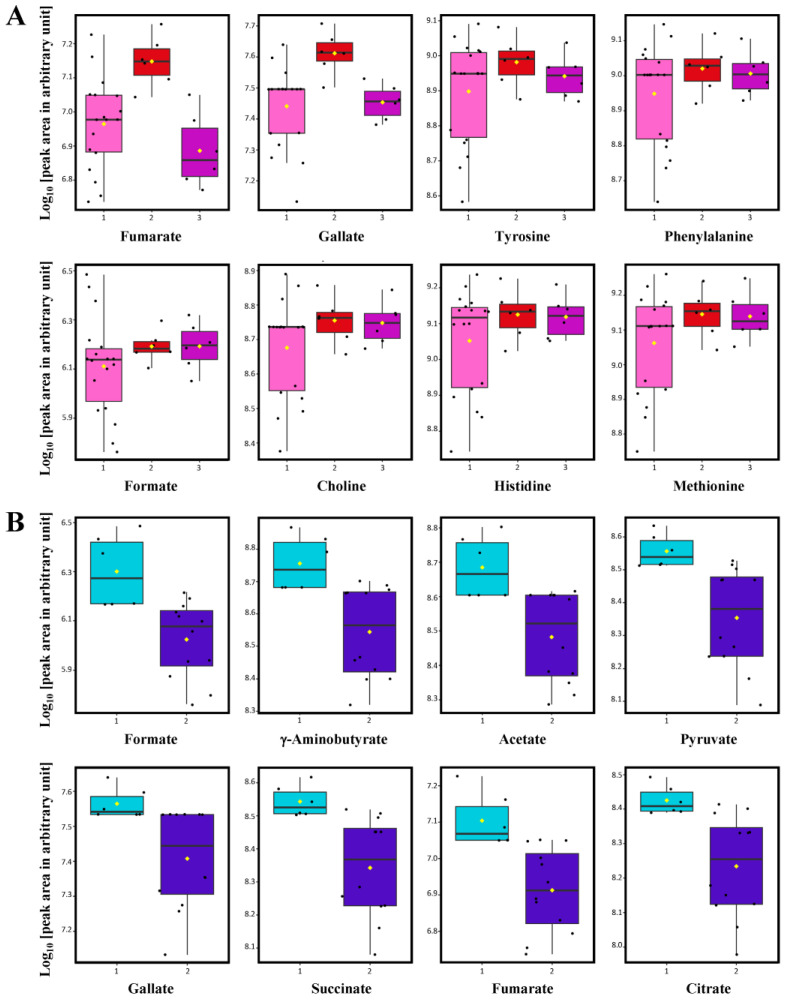
Box-whisker plots represent comparative quantification of potential biomarker metabolites (log10 [peak area of respective compound in arbitrary unit]) responsible for discrimination among Thai Shiraz wine samples aged in new (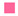), medium-used (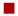) and old used (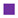) oak wood barrels (**A**) and discrimination between wine samples aged in small (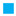) and medium (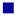) size barrels (**B**). The lower and upper edges of the box denote the 25th and 75th percentile of observation, respectively; the bold line within the box denotes the median value; the yellow rhombus spot (◊) within the box denotes the average value; whiskers denote the 5th and 95th percentiles.

It has been documented that the number of times the barrels are repeatedly used is another key point that considerably influences the wine enrichment capacity during aging [22]. Due to the fact that the pool of oak extractives in a barrel is finite, the quantity of compounds released by oak woods becomes remarkably exhausted with the utilization of the barrel over successive years [22]. Studies on the influences of the repeated use of oak wood barrels are mostly dedicated to the formation of aroma volatiles and biochemical transformation of polyphenolic substances in wines [24,34,35]. It should be noted that little attention has been paid to the impact of barrel utilization time on the variations in non-volatile polar metabolite composition of wines. Therefore, this context was mainly focused on in the present study. The second PLS-DA score plot was constructed with a prediction accuracy of 66.67%, *R*^2^ = 0.625 and *Q*^2^ = 0.520 to evaluate the effect of the repeated use of oak barrels on the metabolite profiles of Shiraz wine samples (*n* = 30) (Figure 2C). Results demonstrated a complete distinction of wine samples aged in old oak barrels (>10 years old) from the ones aged in new (2 years old) and medium-used (6 years old) barrels and the two latter groups were partially overlapped together. VIP scores with a value greater than 1.0 and *p* ≤ 0.05 suggested that variations in the concentration of fumarate, gallate, tyrosine, phenylalanine, formate, choline, histidine and methionine were responsible for the discrimination among Shiraz wine samples aged in wooden barrels with different utilization times (Figure 2D). The integrated ^1^H-NMR signal intensities of these indicative compounds were compared using a box-whisker plot summary (Figure 3A). Results revealed that the concentrations of these metabolites were mostly higher in wine samples aged with medium-used and old oak barrels. 

Fumaric acid is an intermediate metabolite of the tricarboxylic acid cycle which can be extracellularly generated by many fungal strains. Nowadays, fumaric acid can be applied as an additive for wine acidification and inhibition of lactic acid bacteria involved in malolactic fermentation [36]. Gallic acid is considered one of the most important phenolic acids that predominantly contributes to the astringency of red wines [20]. The accumulation of gallic acid during the barrel aging process might be due to hydrolyzed tannins in oak woods gradually dissolving into the wine [32]. It has been reported that organic acids and esters seemed to be present at higher levels in wines aged in repeatedly used oak barrels [24]. An explanation could be due to the lower degree of evaporation taking place in used barrels because of their lower wood porosity as most of the pores have been plugged by previous deposits of mineral salts and color pigments [24]. The significantly higher level of formate, accountable for volatile acidity, in wine samples aged in medium-used and old oak barrels found in our study (Figure 3A) corresponded well with this assumption. Martínez-Gil et al. [37] have also demonstrated that the content of lowmolecular-weight compounds and their oxidation rate were negatively correlated with the amount of oxygen received by the wine or oxygenation during oak barrel aging. These authors found that a significantly higher content of gallic acid as well as other low-molecular-weight phenols remained in red wines after one year of aging in low oxygen transmission rate barrels [37]. Interestingly, our results showed significantly higher levels of fumarate and gallate in Shiraz wines aged in medium-used barrels compared to those found in new barrel and old barrel aging (Figure 3A). It should be noted that differences in the origin and size of barrels also need to be taken into account because these factors have a substantial influence on the extraction of phenolics from oak woods during the initial stage of barrel aging [24]. When comparing barrels with an equal capacity of 2000 L, assuming a similar ratio of wood contact surface area to wine volume, a significantly higher level of gallate was observed in wines aged in medium-used barrels compared to those found in samples aged in the old ones (Figure 3A). This observation was in line with the literature that addressed a positive correlation between using young barrels and the quantity of oak-related phenolics, especially gallic acid, transferred into wines [23]. Therefore, we speculated that the significantly highest level of gallate found in samples aged in medium-used barrels in this study could possibly come from the optimal scenario between the phenolic extraction and degree of oxygenation in this type of aging container.

Amino acids are one of the most important non-volatile metabolites present in grape musts and wines because they play fundamental roles in nutritional support for yeast growth and as metabolic precursors for the formation of wine aromas [38]. The most abundant amino acids present in wines are proline, arginine, glutamine, alanine and γ-aminobutyrate [38]. It is known that variations in amino acid profiles of wines can be attributed to several factors related to the vinification process, such as degradation of grape proteins and addition of N-sources during must pretreatment, metabolic patterns and autolysis in different yeast and bacterial strains, and biochemical changes during wine aging [38]. Our results demonstrated significantly higher levels of tyrosine, phenylalanine, histidine and methionine as potential biomarkers for indication of wine samples aged in medium-used and old oak wood barrels (Figure 3A). As mentioned above, we speculated that the significantly lower contents of amino acids in wines aged with new oak barrels found in this study could possibly be linked to the higher degree of oxygenation taking place in new barrels because of their higher wood porosity [24]. To support this assumption, it has been documented that oxidative reactions of phenolic substances during barrel aging yields quinones which can actively react with several nucleophiles, such as tannins, flavan-3-ols and amino acids, resulting in the reduction of these compounds in the finished wines [39]. Also, oxidative effects on the degradation of sulfur-containing amino acids, such as phenylalanine and methionine, in wines have been reported [40]. From a sensory perspective, it should be mentioned that amino acids are the important flavor-related components in wines because they can be metabolized by yeasts and lactic acid bacteria during fermentation and in the early phase of barrel aging [13]. As a result, certain aroma volatile metabolites derived from amino acid catabolism, mainly higher alcohols and their ester derivatives, have been positively correlated with the desirable flavor characteristics of wines [13]. Concerning the indicative amino acids presented in Figure 3A, tyrosine and phenylalanine are aromatic amino acids and precursors for the biosynthesis of tyrosol and 2-phenylethanol, respectively [41]. These two higher alcohols are derived from yeast amino acid catabolism via the Ehrlich pathway and are known to be associated with the vegetal, honey, floral and rose-like aroma notes in wines [41]. The presence of histidine in wines is connected with nucleotide biosynthesis and catabolism of glutamine and glutamate [42]. Histamine can be converted to histaminol via the Ehrlich pathway by several yeast strains which is now considered as an alternative strategy to reduce the formation of histamine, a biogenic amine that is toxic for humans, in wines [43]. 

Methionine is a sulfur-containing amino acid that can be converted to methional responsible for sulfurous off-flavors, such as cooked potato and a light-struck taste, in wines [40,41]. Therefore, the significantly lower levels of these free amino acids remaining at the end of aging process may suggest a potentially higher aroma complexity in the samples from new oak barrel aging.

### 2.6. Influence of Aging Barrel Volume on the Metabolite Profiles of Wine Samples

It should be mentioned that the use of different capacities of oak wood barrels could also play an important role in the aging process of wines. To evaluate the influence of barrel volume on the metabolite profiles of wines, only samples aged in new oak barrels (*n* = 18) were introduced to another PLS-DA analysis. A score plot was constructed with a prediction accuracy of 77.78%, *R*^2^ = 0.556 and *Q*^2^ = 0.283 (Figure 2E). Results showed a good separation between wine samples aged in small (225 L) and medium (500 L) size barrels. A large variation among samples aged in medium-size barrels could be attributed to the origin and basic properties of oak woods from different barrel-making cooperages (Table 1). VIP scores with a value greater than 1.0 suggested that the concentrations of all indicative metabolites accountable for the discrimination, i.e., formate, γ-aminobutyrate, acetate, pyruvate, gallate, succinate, fumarate, citrate, tartrate and methionine were significantly higher in wine samples aged in small barrels compared to those matured in the larger volume ones (Figure 2F and Figure 3B). Interestingly, it was remarkable that all potential biomarkers were mainly the metabolites in the organic acid class. Indeed, the higher levels of organic acids in wines aged in 225 L barrels were consistent with their higher titratable acidity values compared to those of samples aged in 500 L barrels (Table 1). Although organic acids found in wines are principally derived from grapes and microbial metabolisms during the fermentation process, dynamics in their concentrations during barrel aging have also been mentioned [44]. Our results correspond well with many studies in which the concentration of oak-related aroma volatile substances, total phenolic content and color intensity of wines aged in smaller-size wooden barrels were reported to be significantly higher than those observed in wines aged in the larger volume ones [23,24,45]. The most likely explanation could be that major chemical transformations attributed to the barrel aging process, including evaporation, extraction, oxidation and component reaction, would be intensified with increased wood surface in contact with a unit of wine filled in the barrels, i.e., referred to a higher surface/volume ratio [46]. Although the total phenolic contents between Shiraz wine samples aged in new barrels with 225 and 500 L capacity were not found to be significantly different in this study (Table 1), a good distinction between their metabolite profiles was successfully revealed by ^1^H-NMR combined with chemometric analysis. From a sensory viewpoint, significant contributions of organic acids to the chemical stability and sensory characteristics of wines are well-acknowledged [44,47]. For example, succinic, pyruvic, and lactic acids can be correlated with fresh, sour and metallic notes or even the salty-bitter taste of wines [47]. Acetic and formic acids are accountable for volatile acidity and their excessive abundance can bring a pungent vinegary perception to wines [47]. Moreover, a slightly higher alcoholic strength found in samples aged in small-size barrels (Table 1) could enhance the extraction of oak-related compounds potentially resulting in higher aroma intensity and flavor complexity in this group of wines. 

Collectively, our results support the effectiveness of using a non-targeted ^1^H-NMR metabolomics approach for the molecular characterization of Thai Shiraz wines aged under different conditions. In addition to the existing information on dynamics of phenolic and aroma volatile substances reported in the literature, variations in the non-volatile polar metabolite composition of red wines associated with particular types of aging containers, along with the utilization time and volume of barrels, were successfully revealed in the present study. Understanding the influences of these parameters on wine metabolite formation and stability could support winemakers in order to fine-tune the use of barrels to the desired enrichment capacity. To the authors’ best knowledge, this is the first time that the metabolome of authentic red wines industrially produced in Thailand has been reported. Nevertheless, it should be mentioned that this work was only an exploratory step with a limited number of samples. The reliability of results should be validated with a larger number of wines through a series of vintages. From a short-term perspective, integration of the non-volatile metabolite information with aroma volatile profiles and sensory perception parameters of the wines is another point of our interest that requires further investigations. 

## 3. Materials and Methods

### 3.1. Wine Samples

Shiraz is one of the major grape varieties grown and primarily used to produce high-quality red wines worldwide. This cultivar can also develop and adapt well under sufficient sunlight and the tropical climate conditions of Thailand [48]. Accordingly, red wines produced on an industrial scale from 100% Shiraz grapes harvested in the 2019 vintage were selected in this study. The must was fermented at 20–25 °C for 10–12 days to obtain a dry red wine with an ethanol content of ca. 12%vol. The wines were then allocated into 10 groups based on (i) type of aging containers, i.e., stainless-steel tanks vs. oak wood barrels, (ii) utilization time of oak barrels, i.e., new (2 years old), medium-used (6 years old) and old (>10 years old) and (iii) volume of oak barrels, i.e., small (225 L), medium (500 L) and large (2000 L) size (Table 2). All barrels and tanks were 70–80% filled and stored under a temperature (15–17 °C) and relative humidity (75–85%) controlled condition. Samples were collected after maturation for a period of 18 months. For chemical analysis, three or six replicates of samples were taken from each aging container. It should be noted that wine samples and technical information were kindly provided by Siam Winery Co., Ltd. without any influence on the publication of results.

### 3.2. Determination of pH and Titratable Acidity

The pH of wine samples was determined using a benchtop pH meter (S230, Mettler Toledo, Columbus, OH, USA) with automatic temperature compensation. The total titratable acidity (TA) of wines was determined using an automatic titrator (916 Ti-Touch, Metrohm, Herisau, Switzerland) according to AOAC official method 962.12 [49]. Samples were degassed and titrated with 0.1 N NaOH standard solution (Merck, Darmstadt, Germany) by continuous magnetic stirring until pH 8.20 was reached. The TA value of samples was calculated and expressed as gram of tartaric acid/L [49]. 

### 3.3. Determination of Alcoholic Strength by Volume

The alcoholic strength by volume of wine samples was determined using an NIR-based Alcolyzer Wine ME analysis system combined with a density meter (Anton Paar GmbH, Graz, Austria) according to the manufacturer’s instructions. The alcohol content was calculated, corrected and expressed as percentage of alcohol by volume (%vol).

### 3.4. Determination of Residual Sugar Content

The residual sugar content of wine samples was determined using the 3,5-dinitrosalicylic acid (DNS) (Sigma Aldrich, St. Louis, MO, USA) colorimetric assay [50] combined with UV–Vis spectrophotometry measurement at 540 nm (U-2900/2910, Hitachi, Tokyo, Japan). A calibration curve was constructed using standard solutions of glucose (Merck, Darmstadt, Germany) and fitted by linear regression analysis. The residual sugar content of samples was calculated and expressed as gram of glucose/L. 

### 3.5. Determination of Total Phenolic Content

The total phenolic content of wine samples was determined using the Folin–Ciocalteu (FC) colorimetric assay combined with UV–Vis spectrophotometry measurement at 765 nm (U-2900/2910, Hitachi, Tokyo, Japan) [51]. A calibration curve was constructed using standard solutions of gallic acid (Merck, Darmstadt, Germany) and fitted by linear regression analysis. The total phenolic content of samples was calculated and expressed as mg gallic acid equivalents (GAE)/L.

### 3.6. Sample Preparation and ^1^H-NMR Analysis

Wine samples were prepared according to the method optimized from our previous works [31,32,52] together with the work of Mascellani et al. [26]. In brief, the pH of samples was determined and adjusted to 6.0 using 1.0 N NaOH. Insoluble particles were primarily removed by centrifugation under 4100× *g* at 25 °C for 15 min. The clear wine supernatant was ultrafiltrated through a centrifugal device with 3 kDa molecular weight cut-offs (Pall Nanocep^®^, Pall Life Sciences, Ann Arbor, MI, USA). The filtrate was then diluted 1:1 (*v*/*v*) with phosphate buffer pH 6.0 (300 mM KH_2_PO_4_, 10% (*w*/*w*) D_2_O) consisting of 1 mM 3-(Trimethylsilyl) propionic-2, 2, 3, 3-d_4_ acid sodium salt (TSP) (Merck, Darmstadt, Germany) as an internal standard. Finally, 600 μL of the mixture was transferred to a 5 mm NMR tube and subjected to a Bruker Avance III HD 500 MHz NMR spectrometer (Bruker, Rheinstetten, Germany). The temperature was set to 300 K (26.8 °C). The acquisition was operated at a time domain (TD) of 65,536, an acquisition time (AQ) requirement of 3.198 s, a number of scans (NS) of 256 and a relaxation time (D1) set to 4 s. The Bruker pulse sequence (noesygppr1d) was applied to suppress the residual water signal. 

### 3.7. ^1^H-NMR Spectra Processing and Data Acquisition

^1^H-NMR spectra were phase/baseline corrected and pre-treated as described in our previous work [31] using Topspin software version 3.6.3 (Bruker Biospin, Rheinstetten, Germany). The ^1^H-NMR spectrum (δ = 0.00–10.00 ppm) was segmented using a binning technique with a 0.02 ppm interval. The water region (δ = 4.60–4.80) was removed from the analysis. Identification of metabolites was performed by means of Chenomx NMR suite 9.0 library (Chenomx Inc., Edmond, AB, Canada), Food Metabolite Database (https://foodb.ca/; accessed on 31 January 2023) and the relevant literature [25,26]. The sum of signal intensities (arbitrary units) from all bins accountable for respective metabolites was introduced to statistical analysis [52]. 

### 3.8. Statistical Analysis

Analysis of variance (ANOVA) combined with multiple comparisons by Duncan’s test was performed using SPSS statistical package ver. 28.0 (SPSS Inc., Chicago, IL, USA). A significantly different level was considered at *p* ≤ 0.05. ^1^H-NMR metabolomic data were normalized before subjecting them to multivariate analysis [31]. Heatmap visualization combined with Pearson’s correlation-based hierarchical clustering (HCA) and partial least-squares discriminant analysis (PLS-DA) was performed using Multi-Experiment Viewer “http://mev.tm4.org (accessed on 25 April 2023)” and MetaboAnalyst “www.metaboanalyst.ca (accessed on 25 April 2023)” 5.0 software, respectively. The quality of the PLS-DA model was expressed by *R*^2^ (accuracy) and *Q*^2^ values (predictability) derived from a leave-one-out cross-validation (LOOCV) test. Finally, metabolites with variable importance in projection (VIP) score > 1.0 and *p* ≤ 0.05 were considered to be potential biomarkers responsible for the discrimination [31].

## 4. Conclusions

The present study demonstrates an application of non-targeted ^1^H-NMR metabolomics for the molecular characterization of Shiraz wine samples aged under different conditions. A total of 37 non-volatile polar metabolites were identified using a high-resolution ^1^H-NMR technique. Chemometric analysis revealed that the metabolite profiles of wine samples could be successfully distinguished, according to different types of aging containers, as well as the utilization times and volumes of oak wood barrels used in the maturation process. A set of indicative metabolites consisting of organic acids, amino acids and phenolic acids were statistically suggested as potential biomarkers of which the abundances were significantly altered by the oak barrel-associated factors investigated. Variations in the concentration of these metabolites would have significant impacts on the organoleptic characteristics of finished wine products. This information could be further applied to support the decisions of winemakers in order to fine-tune the use of aging containers and manipulate maturation techniques to achieve the desired wine quality and style.

## Figures and Tables

**Table 1:** Major chemical characteristics of wine samples.

Type of Aging Container	Sample Code	pH	Titratable Acidity (g Tartaric Acid/L)	Alcohol (%vol)	Residual Sugar (g Glucose/L)	Total Phenolics (mg GAE/L)
New oak	O-RD-G7 (225 L)	3.68 * ± 0.02 ^c^**	6.80 ± 0.02 ^f^	14.08 ± 0.02 ^d^	7.44 ± 0.76 ^c^	2579.44 ± 39.38 ^d^
	O-RD-G5 (225 L)	3.62 ± 0.01 ^b^	6.60 ± 0.02 ^e^	15.54 ± 0.01 ^g^	6.82 ± 0.45 ^bc^	2646.11 ± 41.11 ^d^
	O-RD-A18 (500 L)	3.65 ± 0.02 ^b^	6.53 ± 0.02 ^d^	13.90 ± 0.01 ^cd^	6.12 ± 0.32 ^ab^	2287.78 ± 25.46 ^bc^
	O-SM-A3 (500 L)	3.61 ± 0.02 ^ab^	6.15 ± 0.02 ^a^	14.24 ± 0.02 ^e^	6.97 ± 0.30 ^bc^	2585.00 ± 80.36 ^d^
	O-SV-A8 (500 L)	3.60 ± 0.01 ^ab^	6.21 ± 0.01 ^b^	14.28 ± 0.01 ^e^	6.86 ± 0.33 ^bc^	2626.67 ± 72.65 ^d^
	O-TS-A12 (500 L)	3.69 ± 0.02 ^c^	6.26 ± 0.02 ^b^	13.84 ± 0.01 ^c^	7.00 ± 0.35 ^bc^	2673.89 ± 119.70 ^d^
Medium-used oak	O-RD-E6 (2000 L)	3.67 ± 0.01 ^c^	6.64 ± 0.02 ^e^	13.29 ± 0.02 ^a^	6.35 ± 0.38 ^abc^	2537.78 ± 62.55 ^d^
Old oak	O-FF-FF1 (2000 L)	3.55 ± 0.02 ^a^	6.43 ± 0.02 ^c^	13.32 ± 0.01 ^a^	5.35 ± 0.12 ^a^	1582.22 ± 67.55 ^a^
	O-AB-ASS1 (2000 L)	3.66 ± 0.02 ^bc^	6.25 ± 0.01 ^b^	14.42 ± 0.01 ^f^	6.25 ± 0.62 ^abc^	2326.67 ± 25.00 ^c^
Stainless steel	SS (2000 L)	3.59 ± 0.02 ^a^	6.18 ± 0.02 ^ab^	13.42 ± 0.01 ^b^	6.13 ± 0.17 ^ab^	2112.78 ± 62.55 ^b^

* Values are the average of samples from three or six replicates ± SD. ** Letters (a–g) indicate significant difference (*p* ≤ 0.05) among mean values within the same column.

**Table 2:** Information on Shiraz wine samples used in this study.

Group	Sample Code	Replicate	Type of Aging Container	Brand of Barrels *	Age of Barrels **	Container Size ***
1	SS	6	Stainless steel	NA	NA	Large
2	O-RD-G7	3	Oak	A	New	Small
3	O-RD-G5	3	Oak	A	New	Small
4	O-RD-A18	3	Oak	A	New	Medium
5	O-RD-E6	6	Oak	A	Medium	Large
6	O-SM-A3	3	Oak	B	New	Medium
7	O-SV-A8	3	Oak	C	New	Medium
8	O-TS-A12	3	Oak	D	New	Medium
9	O-FF-FF1	3	Oak	E	Old	Large
10	O-AB-ASS1	3	Oak	F	Old	Large

NA = not applicable; * Brands of barrels are preferably not mentioned. ** Age of oak barrels: New = 2, medium = 6, old = >10 years old. *** Size of oak barrels: Small = 225 L, medium = 500 L, Large = 2000 L.

## Data Availability

Data produced in this study can be available from the corresponding author on a reasonable request.

## Supplementary Materials

The following supporting information can be downloaded at: https://www.mdpi.com/article/10.3390/molecules28186716/s1, Figure S1. Representative NOESY-1D-^1^H-NMR spectra of a Shiraz wine sample (panel A) and expansions corresponding for aliphatic region (panel B), sugar region (panel C) and aromatic region (panel D) with assigned peaks; Table S1. Assignment table of the non-volatile polar metabolites present in the ^1^H-NMR spectra of Thai Shiraz wine sample; Table S2. Comparative quantification of non-volatile polar metabolites identified in Thai Shiraz wine samples using high-resolution NOESY-1D-^1^H-NMR spectroscopy (500 MHz).
