# Supplementary material for: Influence of Different Types, Utilization Times, and Volumes of Aging Barrels on the Metabolite Profile of Red Wine Revealed by 1H-NMR Metabolomics Approach
Source: Molecules. 2023 Sep 20;28(18):6716. doi: 10.3390/molecules28186716 (PMC10534683; doi:10.3390/molecules28186716)
Supplement: Supplementary file 1 [file molecules-28-06716-s001.zip › molecules-2599304-supplementary.pdf]

Supplementary Materials

Article

Influence of Different Types, Utilization Times, and Volumes of Aging Barrels on the Metabolite Profile of Red Wine Revealed by ^{1}H-NMR Metabolomics Approach

Suwanan Denchai [1,2], Suppached Sasomsin [3], Cheunjit Prakitchaiwattana [2], Thanitaporn Phuenpong [2], Kunaporn Homyog [4], Wanwimon Mekboonsonglarp [5] and Sarn Settachaimongkon [2,6,7,*]

1 Program in Biotechnology, Faculty of Science, Chulalongkorn University, Bangkok 10330, Thailand; 6370195223@student.chula.ac.th
2 Department of Food Technology, Faculty of Science, Chulalongkorn University, Bangkok 10330, Thailand
3 Innovation & Winemaking Division, Siam Winery Company Limited, Samut Sakhon 74000, Thailand; suppached.s@siamwinery.com
4 Center of Veterinary Diagnosis, Faculty of Veterinary Science, Mahidol University, Nakhon Pathom 73170, Thailand
5 Scientific and Technological Research Equipment Center (STREC), Chulalongkorn University, Bangkok 10330, Thailand
6 Emerging Processes for Food Functionality Design Research Unit, Chulalongkorn University, Bangkok 10330, Thailand
7 Omics Sciences and Bioinformatics Center, Faculty of Science, Chulalongkorn University, Bangkok 10330, Thailand
* Correspondence: sarn.s@chula.ac.th

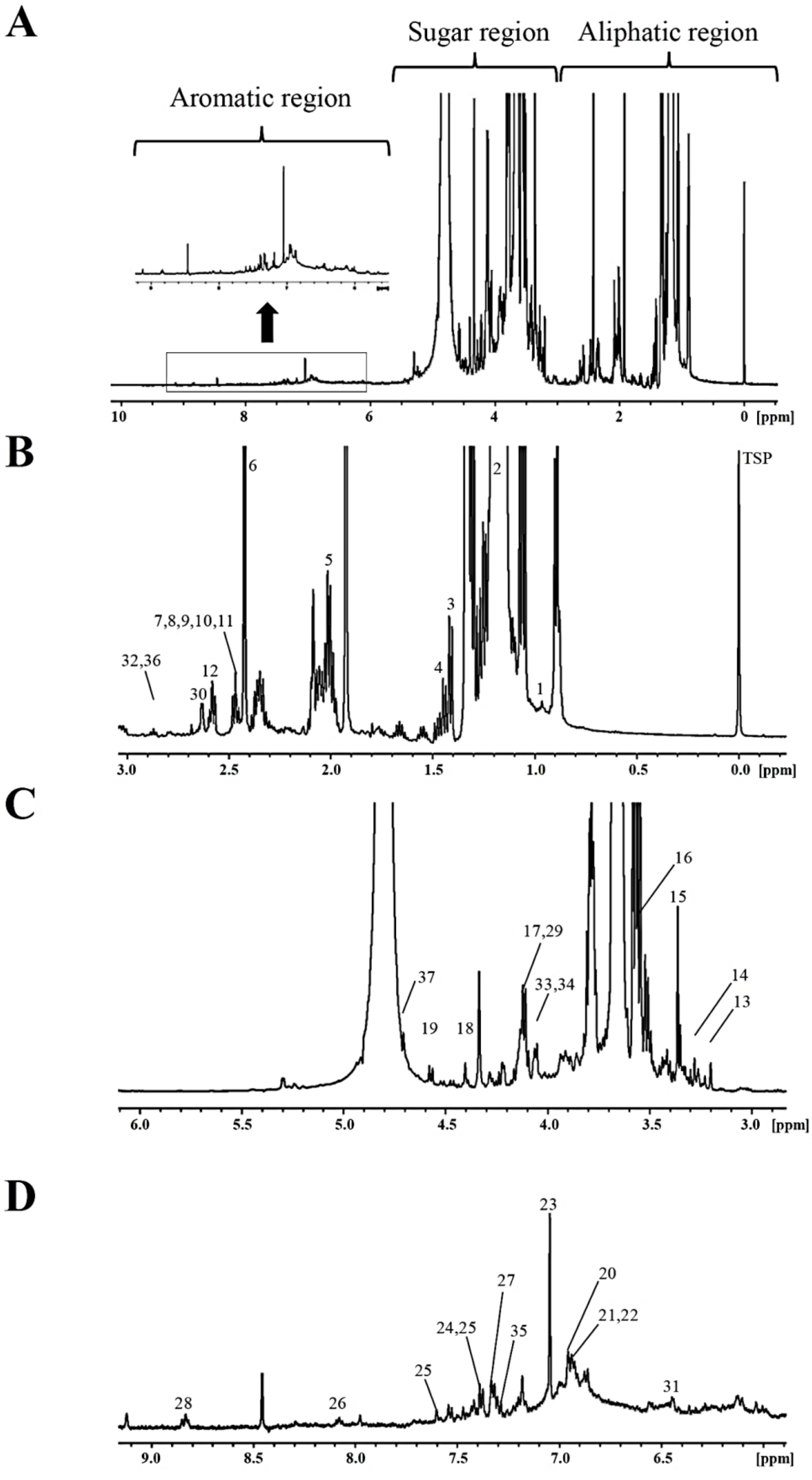
A
Sugar region
Aliphatic region
Aromatic region
10 8 6 4 2 0 [ppm]
B
6
2
TSP
5
3
7,8,9,10,11
32,36
12
30
4
1
3.0 2.5 2.0 1.5 1.0 0.5 0.0 [ppm]
C
16
15
17,29
37
33,34
14
13
19
18
6.0 5.5 5.0 4.5 4.0 3.5 3.0 [ppm]
D
23
27
20
24,25
35
21,22
25
28
26
31
9.0 8.5 8.0 7.5 7.0 6.5 [ppm]

Figure S1. Representative NOESY-1D-^{1}H-NMR spectra of a Shiraz wine sample (panel A) and expansions corresponding for aliphatic region (panel B), sugar region (panel C) and aromatic region (panel D) with assigned peaks: TSP : internal standard, 1: leucine, 2: ethanol, 3: lactate, 4: alanine, 5: proline, 6: acetate, 7: methionine, 8: acetoin, 9: acetoacetate, 10: pyruvate, 11: glutamate, 12: succinate, 13: choline, 14: myo-inositol, 15: methanol, 16: glycerol, 17: fructose, 18: tartrate, 19: glucose, 20: epicatechin, 21: *p*-hydroxyphenyl acetate, 22: tyrosine, 23: gallate, 24: phenylalanine, 25: chlorogenate, 26: formate, 27: histidine, 28: trigonelline, 29: arginine, 30: citrate, 31: fumarate, 32: γ-aminobutyrate, 33: malate, 34: mannitol, 35: syringate, 36: threonine and 37: valine.

Table S1. Assignment table of the non-volatile polar metabolites present in the ^{1}H-NMR spectra of Thai Shiraz wine sample

Chemical Group	Metabolite	Chemical Shift (ppm) [a]
Alcohols	(2) Ethanol	1.17 (t) [b] 3.65 (q)
	(16) Glycerol	3.55 (m) 3.64 (m) 3.65 (m)
	(34) Mannitol	3.66 (m) 3.76 (m) 3.78 (d) 3.83 (m)
	(15) Methanol	3.35 (s)
	(14) Myo-Inositol	3.26 (t) 3.52 (m) 3.61 (t) 4.05 (t)
Amino acids	(4) Alanine	1.47 (d) 3.77 (q)
	(29) Arginine	3.25 (t) 3.77 (t)
	(11) Glutamate	2.13 (m) 2.32 (t) 2.37 (t) 3.76 (m)
	(27) Histidine	3.24 (m) 3.31 (m) 4.00 (m) 7.27 (s)
	(1) Leucine	0.95 (m) 3.74 (m)
	(7) Methionine	2.12 (s) 2.63 (t) 3.87 (t)
	(24) Phenylalanine	3.15 (m) 3.28 (m) 4.00 (m) 7.31 (d) 7.36 (m) 7.40 (m) 7.41 (m)
	(5) Proline	1.99 (m) 2.03 (m) 2.06 (m) 2.34 (m) 3.33 (m) 3.41 (m)
	(36) Threonine	1.32 (d) 3.60 (d) 4.26 (m)
	(22) Tyrosine	3.04 (m) 3.19 (m) 3.94 (m) 6.89 (d) 7.18 (d)
	(37) Valine	0.96 (d) 1.04 (d) 2.27 (m) 3.60 (d)
Organic acids	(6) Acetate	1.91(s)
	(9) Acetoacetate	2.27 (s) 3.44 (s)
	(8) Acetoin	2.21 (s) 4.43 (m)
	(13) Choline	3.19 (s) 4.05 (m)
	(30) Citrate	2.55 (d) 2.70 (d)
	(26) Formate	8.44 (s)
	(31) Fumarate	6.53 (s)
	(32) γ-Aminobutyrate	1.89 (m) 2.29 (t) 3.00 (t)
	(3) Lactate	1.32 (d) 4.10 (m)
	(33) Malate	2.40 (m) 2.68 (m) 4.30 (d)
	(10) Pyruvate	2.36 (s)
	(12) Succinate	2.41 (s)
	(18) Tartrate	4.32 (s)
	(28) Trigonelline	4.42 (s) 8.06 (t) 8.81 (d) 8.83 (d) 8.84 (d) 9.11 (s)
Phenolic compounds	(25) Chlorogenate	2.01 (t) 2.03 (t) 3.88 (m) 4.26 (d) 6.94 (d) 7.18 (d)
	(20) Epicatechin	2.76 (d) 2.93 (m) 4.35 (s) 5.00 (s) 6.95 (t) 7.04 (s)
	(23) Gallate	7.03 (s)
	(21) *p*-Hydroxyphenyl-acetate	3.44 (s) 6.85 (d) 7.16 (d)
	(35) Syringate	3.89 (s) 7.27 (s)
Sugars	(19) Glucose	3.24 (t) 3.40 (m) 3.45 (m) 3.48 (m) 3.53 (m) 3.70 (m) 3.71 (m) 3.76 (m) 3.80 (m) 3.83 (m) 3.88 (m)
	(17) Fructose	3.55 (m) 3.58 (m) 3.66 (m) 3.70 (m) 3.78 (m) 3.80 (m) 3.89 (m) 4.00 (m) 4.11 (d)

[a] Chemical shift values are referenced to TSP signal (0.00 ppm) at pH 6.00.

[b] Letters indicate singlet (s), doublet (d), triplet (t) and multiplet (m) multiplicity of ^{1}H-NMR peaks, respectively.

Table S2. Comparative quantification of non-volatile polar metabolites identified in Thai Shiraz wine samples using a high resolution NOESY-1D-^{1}H-NMR spectroscopy (500 MHz). Metabolite contents are expressed as log$_{10}$ [peak area of respective compound in arbitrary unit]. Values are the average from three or six replicates of samples corresponding to the respective type of aging containers. Superscript letters (a-f) indicate significant difference ($p \leq 0.05$) among sample means within the same row.

Chemical Group	Metabolite Name	New oak						Medium oak	Old oak		Stainless steel
		ORD-G7	ORD-G5	ORD-A18	OSM-A3	OSV-A8	OTS-A12	ORD-E6	OFF-FF1	OAB-ASS1	SS
Alcohol	Ethanol	10.40±0.03^{d}	10.36±0.00^{c,d}	10.08±0.10^{a}	10.36±0.01^{c,d}	10.11±0.04^{a}	10.29±0.00^{b,c}	10.37±0.07^{c,d}	10.39±0.07^{d}	10.37±0.05^{c,d}	10.27±0.04^{b}
	Glycerol	10.39±0.03^{d}	10.35±0.00^{c,d}	10.08±0.10^{a}	10.36±0.01^{c,d}	10.11±0.04^{a}	10.28±0.00^{b,c}	10.37±0.07^{c,d}	10.38±0.07^{d}	10.36±0.05^{c,d}	10.26±0.04^{b}
	Mannitol	10.40±0.03^{d}	10.35±0.00^{c,d}	10.08±0.010^{a}	10.36±0.01^{c,d}	10.11±0.04^{a}	10.28±0.00^{b,c}	10.37±0.07^{c,d}	10.39±0.07^{d}	10.37±0.05^{c,d}	10.27±0.04^{b}
	Methanol	8.73±0.03^{c}	8.66±0.00^{b,c}	8.36±0.09^{a}	8.68±0.01^{b,c}	8.43±0.04^{a}	8.64±0.00^{b,c}	8.65±0.07^{b,c}	8.68±0.07^{b,c}	8.61±0.05^{b}	8.60±0.04^{b}
	Myo-Inositol	10.42±0.03^{d}	10.37±0.00^{c,d}	10.09±0.10^{a}	10.38±0.01^{c,d}	10.13±0.04^{a}	10.30±0.00^{b,c}	10.39±0.07^{c,d}	10.40±0.07^{d}	10.38±0.05^{c,d}	10.28±0.04^{b}
Amino acid	Alanine	10.41±0.03^{d}	10.37±0.00^{c,d}	10.09±0.10^{a}	10.38±0.01^{c,d}	10.13±0.04^{a}	10.30±0.00^{b,c}	10.38±0.07^{c,d}	10.40±0.07^{d}	10.38±0.05^{c,d}	10.28±0.04^{b}
	Arginine	10.33±0.04^{d}	10.29±0.00^{c,d}	10.01±0.10^{a}	10.30±0.01^{c,d}	10.04±0.05^{a}	10.22±0.00^{b,c}	10.30±0.06^{c,d}	10.32±0.07^{d}	10.29±0.05^{c,d}	10.20±0.04^{b}
	Glutamate	10.33±0.04^{d}	10.29±0.00^{c,d}	10.01±0.10^{a}	10.30±0.01^{c,d}	10.04±0.05^{a}	10.22±0.00^{b,c}	10.30±0.06^{c,d}	10.32±0.07^{d}	10.29±0.05^{c,d}	10.20±0.04^{b}
	Histidine	9.20±0.03^{d}	9.14±0.00^{b,c,d}	8.83±0.09^{a,c}	9.15±0.01^{c,d}	8.89±0.04^{a}	9.10±0.00^{b}	9.13±0.07^{b,c,d}	9.14±0.08^{b,c,d}	9.10±0.05^{b,c}	9.05±0.04^{b}
	Leucine	10.40±0.03^{d}	10.36±0.00^{c,d}	10.08±0.10^{a}	10.36±0.01^{c,d}	10.11±0.05^{a}	10.28±0.00^{b,c}	10.37±0.07^{c,d}	10.39±0.07^{d}	10.37±0.05^{c,d}	10.27±0.04^{b}
	Methionine	9.22±0.04^{c}	9.11±0.00^{b}	8.84±0.09^{a}	9.17±0.01^{b,c}	8.91±0.04^{a}	9.11±0.00^{b}	9.15±0.07^{b,c}	9.18±0.07^{b,c}	9.10±0.05^{b}	9.09±0.04^{b}
	Phenylalanine	9.11±0.04^{d}	9.00±0.00^{b,c}	8.73±0.09^{a}	9.05±0.01^{d}	8.80±0.04^{a}	9.00±0.00^{b,c}	9.02±0.07^{b,c}	9.03±0.07^{b,c,d}	8.98±0.05^{b,c}	8.95±0.04^{b}
	Proline	10.43±0.03^{d}	10.39±0.00^{c,d}	10.11±0.10^{a}	10.40±0.01^{c,d}	10.14±0.04^{a}	10.32±0.00^{b,c}	10.40±0.07^{c,d}	10.42±0.07^{d}	10.40±0.05^{c,d}	10.30±0.04^{b}
	Threonine	10.75±0.03^{d}	10.71±0.00^{c,d}	10.43±0.10^{a}	10.72±0.10^{c,d}	10.47±0.04^{a}	10.64±0.00^{b,c}	10.72±0.07^{c,d}	10.75±0.07^{d}	10.73±0.05^{d}	10.62±0.04^{b}
	Tyrosine	9.05±0.04^{d}	8.95±0.04^{b,c}	8.67±0.00^{a}	9.01±0.01^{c,d}	8.75±0.04^{a}	8.95±0.00^{b,c}	8.98±0.07^{b,c,d}	8.96±0.08^{b,c,d}	8.92±0.05^{b,c}	8.89±0.04^{b}
	Valine	10.82±0.03^{d}	10.78±0.00^{c,d}	10.50±0.10^{a}	10.79±0.01^{c,d}	10.53±0.04^{a}	10.71±0.00^{b,c}	10.79±0.07^{c,d}	10.81±0.07^{d}	10.73±0.05^{c,d}	10.69±0.04^{b}
Organic acid	Acetate	8.76±0.04^{d}	8.60±0.00^{b,c}	8.37±0.08^{a}	8.60±0.01^{b,c}	8.35±0.03^{a}	8.60±0.00^{b,c}	8.66±0.07^{b,c}	8.67±0.07^{c}	8.59±0.05^{b,c}	8.57±0.04^{b}
	Acetoacetate	10.34±0.04^{d}	10.30±0.00^{b,c,d}	10.02±0.10^{a}	10.31±0.01^{c,d}	10.05±0.05^{a}	10.23±0.00^{b,c}	10.32±0.07^{c,d}	10.34±0.08^{d}	10.32±0.05^{c,d}	10.22±004^{b}
	Acetoin	8.62±0.04^{e}	8.49±0.00^{c,d}	8.11±0.05^{a}	8.49±0.00^{c,d}	8.31±0.04^{b}	8.50±0.00^{c,d}	8.44±0.07^{c}	8.56±0.07^{d,e}	8.56±0.05^{d,e}	8.46±0.05^{c}
	Choline	8.85±0.04^{c}	8.74±0.00^{b}	8.46±0.08^{a}	8.74±0.09^{b}	8.53±0.04^{a}	8.74±0.00^{b}	8.76±0.07^{b}	8.77±0.07^{b,c}	8.72±0.05^{b}	8.68±0.04^{b}
	Citrate	8.46±0.04^{e}	8.39±0.00^{c,d,e}	8.05±0.07^{a}	8.40±0.01^{c,d,e}	8.15±0.03^{b}	8.33±0.00^{c,d}	8.42±0.08^{d,e}	8.40±0.07^{c,d,e}	8.46±0.05^{e}	8.32±0.05^{c}

Chemical Group	Metabolite Name	New oak						Medium oak	Old oak		Stainless steel
		**ORD-G7**	**ORD-G5**	**ORD-A18**	**OSM-A3**	**OSV-A8**	**OTS-A12**	**ORD-E6**	**OFF-FF1**	**OAB-ASS1**	**SS**
	Formate	6.43±0.05^{e}	6.15±0.00^{b}	5.91±0.15^{a}	6.12±0.02^{b}	5.87±0.07^{a}	6.19±0.03^{b,c}	6.19±0.06^{b,c}	6.12±0.07^{b}	6.27±0.06^{c}	5.91±0.04^{a}
	Fumarate	7.16±0.07^{e}	6.98±0.00^{b,c}	6.88±0.13^{a,b}	7.05±0.00^{c,d}	6.79±0.04^{a}	6.93±0.05^{b}	7.15±0.07^{d,e}	6.97±0.08^{b,c}	6.80±0.03^{a}	6.92±0.04^{b}
	γ-Aminobutyrate	8.83±0.04^{d}	8.67±0.00^{b,c}	8.39±0.07^{a}	8.67±0.01^{b,c}	8.43±0.03^{a}	8.66±0.00^{b,c}	8.71±0.08^{b,c}	8.75±0.08^{c,d}	8.67±0.05^{b,c}	8.64±0.05^{b,c}
	Lactate	10.77±0.03^{d}	10.73±0.00^{c,d}	10.45±0.10^{a}	10.74±0.01^{c,d}	10.49±0.04^{a}	10.66±0.00^{b,c}	10.74±0.07^{c,d}	10.76±0.07^{d}	10.75±0.05^{c,d}	10.64±0.04^{b}
	Malate	9.16±0.04^{e}	9.04±0.00^{c,d}	8.73±0.07^{a}	9.09±0.01^{d,e}	8.84±0.04^{b}	9.03±0.07^{c,d}	9.04±0.07^{c,d}	9.10±0.07^{d,e}	9.09±0.05^{c,d,e}	9.00±0.04^{c}
	Pyruvate	8.60±0.04^{d}	80.52±0.00^{c,d}	8.16±0.07^{a}	8.52±0.01^{c,d}	8.26±0.03^{b}	8.47±0.00^{c}	8.51±0.08^{c,d}	8.55±0.07^{c,d}	8.52±0.05^{c,d}	8.47±0.05^{c}
	Succinate	8.58±0.04^{d}	8.51±0.00^{c,d}	8.16±0.07^{a}	8.51±0.01^{c,d}	8.26±0.03^{b}	8.45±0.00^{c}	8.51±0.08^{c,d}	8.53±0.07^{c,d}	8.52±0.05^{c,d}	8.45±0.05^{c}
	Tartrate	8.52±0.04^{f}	8.39±0.05^{c,d,e}	7.07±0.07^{a}	8.39±0.00^{c,d,e}	8.21±0.04^{b}	8.39±0.00^{c,d,e}	8.38±0.07^{c,d}	8.46±0.07^{d,e,f}	8.46±0.07^{e,f}	8.48±0.05^{c}
	Trigonelline	8.48±0.04^{e}	8.37±0.00^{c,d}	8.02±0.06^{a}	8.45±0.02^{d,e}	8.20±0.04^{b}	8.37±0.00^{c,d}	8.32±0.08^{c}	8.43±0.07^{d,e}	8.45±0.05^{d,e}	8.33±0.05^{c}
Phenolic compounds	Chlorogenate	10.43±0.04^{d}	10.38±0.00^{c,d}	10.10±0.01^{a}	10.10±0.10^{c,d}	10.14±0.04^{a}	10.31±0.00^{b,c}	10.39±0.07^{c,d}	10.41±0.07^{c,d}	10.39±0.05^{c,d}	10.29±0.04^{b}
	Epicatechin	8.98±0.05^{b}	8.93±0.00^{b}	8.70±0.11^{a}	8.88±0.00^{b}	8.70±0.06^{a}	8.86±0.02^{b}	8.84±0.17^{a,b}	8.95±0.07^{b}	8.89±0.05^{b}	8.86±0.03^{b}
	Gallate	7.60±0.05^{c}	7.50±0.00^{b}	7.25±0.11^{a}	7.50±0.00^{b}	7.31±0.04^{a}	7.50±0.00^{b}	7.61±0.07^{c}	7.50±0.07^{b}	7.45±0.05^{b}	7.42±0.04^{b}
	p-Hydroxyphenyl acetate	8.89±0.03^{d}	8.83±0.00^{b,c,d}	8.54±0.09^{a}	8.85±0.01^{c,d}	8.59±0.04^{a}	8.79±0.00^{b,c}	8.84±0.07^{b,c,d}	8.83±0.08^{b,c,d}	8.81±0.05^{b,c,d}	8.75±0.04^{b}
	Syringate	9.10±0.04^{d}	9.05±0.00^{b,c,d}	8.76±0.10^{a}	9.06±0.01^{c,d}	8.81±0.04a	8.99±.0.00^{b,c}	9.05±0.07^{c,d}	9.04±0.07^{b,c,d}	9.00±0.05^{b,c}	8.96±0.04^{b}
Sugar	Glucose	10.41±0.03^{d}	10.36±0.00^{c,d}	10.09±0.10^{a}	10.37±0.01^{c,d}	10.12±0.04^{a}	10.29±0.00^{b,c}	10.38±0.07^{c,d}	10.39±0.07^{d}	10.37±0.05^{c,d}	10.27±0.04^{b}
	Fructose	10.41±0.03^{d}	10.37±0.00^{c,d}	10.09±0.10^{a}	10.37±0.01^{c,d}	10.12±0.04^{a}	10.29±0.00^{b,c}	10.38±0.07^{c,d}	10.40±0.07^{d}	10.37±0.05^{c,d}	10.28±0.04^{b}
